# Supplementary material for: A Pair of CoII Supramolecular Isomers Based on Flexible Bis-Pyridyl-Bis-Amide and Angular Dicarboxylate Ligands
Source: Molecules. 2020 Jan 3;25(1):201. doi: 10.3390/molecules25010201 (PMC6982743; doi:10.3390/molecules25010201)

checkCIF/PLATON report

Structure factors have been supplied for datablock(s) 1, 2

THIS REPORT IS FOR GUIDANCE ONLY. IF USED AS PART OF A REVIEW PROCEDURE FOR PUBLICATION, IT SHOULD NOT REPLACE THE EXPERTISE OF AN EXPERIENCED CRYSTALLOGRAPHIC REFEREE.

No syntax errors found. CIF dictionary Interpreting this report

Datablock: 1

```
Bond precision:  C-C = 0.0027 A         Wavelength=0.71073

Cell:          a=9.1273(2)       b=12.0439(2)      c=12.0526(2)
               alpha=62.4993(8)  beta=75.9211(10)  gamma=79.9212(9)
Temperature:   296 K

                 Calculated                 Reported
Volume           1136.88(4)                 1136.88(4)
Space group      P -1                       P -1
Hall group       -P 1                       -P 1
Moiety formula   C23 H19 Co N2 O7 S         ?
Sum formula      C23 H19 Co N2 O7 S         C23 H19 Co N2 O7 S
Mr               526.39                     526.39
Dx,g cm-3        1.538                      1.538
Z                2                          2
Mu (mm-1)        0.895                      0.895
F000             540.0                      540.0
F000'            541.19
h,k,lmax         12,16,16                   12,16,16
Nref             5663                       5647
Tmin,Tmax        0.732,0.914                0.677,0.746
Tmin'            0.692

Correction method= # Reported T Limits: Tmin=0.677 Tmax=0.746
AbsCorr = MULTI_SCAN

Data completeness= 0.997          Theta(max)= 28.317

R(reflections)= 0.0293( 5131)     wR2(reflections)= 0.0815( 5647)

S = 1.038                  Npar= 307
```

The following ALERTS were generated. Each ALERT has the format
test-name_ALERT_alert-type_alert-level.
Click on the hyperlinks for more details of the test.

Alert level G

```
PLAT004_ALERT_5_G Polymeric Structure Found with Maximum Dimension          1 Info
PLAT007_ALERT_5_G Number of Unrefined Donor-H Atoms ..............          1 Report
PLAT232_ALERT_2_G Hirshfeld Test Diff (M-X)  Co       --O6_a     .        6.8 s.u.
PLAT232_ALERT_2_G Hirshfeld Test Diff (M-X)  Co       --O7_a     .       20.3 s.u.
PLAT764_ALERT_4_G Overcomplete CIF Bond List Detected (Rep/Expd) .       1.17 Ratio
PLAT794_ALERT_5_G Tentative Bond Valency for Co        (II)      .       1.89 Info
PLAT804_ALERT_5_G Number of ARU-Code Packing Problem(s) in PLATON           1 Info
PLAT883_ALERT_1_G No Info/Value for _atom_sites_solution_primary .     Please Do !
PLAT912_ALERT_4_G Missing # of FCF Reflections Above STh/L=  0.600         16 Note
PLAT978_ALERT_2_G Number C-C Bonds with Positive Residual Density.         15 Info
```

```
   0 ALERT level A = Most likely a serious problem - resolve or explain
   0 ALERT level B = A potentially serious problem, consider carefully
   0 ALERT level C = Check. Ensure it is not caused by an omission or oversight
  10 ALERT level G = General information/check it is not something unexpected

   1 ALERT type 1 CIF construction/syntax error, inconsistent or missing data
   3 ALERT type 2 Indicator that the structure model may be wrong or deficient
   0 ALERT type 3 Indicator that the structure quality may be low
   2 ALERT type 4 Improvement, methodology, query or suggestion
   4 ALERT type 5 Informative message, check
```

Datablock: 2

```
Bond precision:  C-C = 0.0072 A          Wavelength=0.71073

Cell:          a=9.2206(8)       b=11.0642(10)     c=13.3450(12)
               alpha=96.611(5)   beta=109.696(5)   gamma=103.697(5)
Temperature:   296 K
```

	Calculated	Reported
Volume	1216.7(2)	1216.73(19)
Space group	P -1	P -1
Hall group	-P 1	-P 1
Moiety formula	2(C23 H19 Co N2 O7 S), C H3 O, 2(H O0.50)	?
Sum formula	C47 H43 Co2 N4 O16 S2	C23.50 H22 Co N2 O8 S
Mr	1101.83	551.42
Dx,g cm-3	1.504	1.505
Z	1	2
Mu (mm-1)	0.842	0.842
F000	567.0	568.0
F000’	568.21	
h,k,lmax	11,13,16	11,13,16
Nref	4805	4661
Tmin,Tmax	0.845,0.959	0.641,0.746
Tmin’	0.845	

```
Correction method= # Reported T Limits: Tmin=0.641 Tmax=0.746
AbsCorr = MULTI_SCAN

Data completeness= 0.970          Theta(max)= 25.999

R(reflections)= 0.0573( 3190)     wR2(reflections)= 0.1531( 4661)

S = 1.024                Npar= 321
```

The following ALERTS were generated. Each ALERT has the format
test-name_ALERT_alert-type_alert-level.
Click on the hyperlinks for more details of the test.

Alert level C

```
PLAT029_ALERT_3_C _diffrn_measured_fraction_theta_full value Low .      0.972 Why?
PLAT041_ALERT_1_C Calc. and Reported SumFormula    Strings  Differ     Please Check
PLAT202_ALERT_3_C Isotropic non-H Atoms in Anion/Solvent .........          1 Check
              O8
PLAT230_ALERT_2_C Hirshfeld Test Diff for    C3       --C4       .        5.5 s.u.
PLAT241_ALERT_2_C High   'MainMol' Ueq as Compared to Neighbors of         O2 Check
PLAT242_ALERT_2_C Low    'MainMol' Ueq as Compared to Neighbors of         C7 Check
PLAT242_ALERT_2_C Low    'MainMol' Ueq as Compared to Neighbors of        C17 Check
PLAT309_ALERT_2_C Single Bonded Oxygen (C-O > 1.3 Ang) ...........         O8 Check
PLAT334_ALERT_2_C Small Aver. Benzene C-C Dist C17      -C22             1.37 Ang.
PLAT341_ALERT_3_C Low Bond Precision on  C-C Bonds ...............    0.00718 Ang.
PLAT413_ALERT_2_C Short Inter XH3 .. XHn     H2B      ..H24A     .       2.00 Ang.
                                                1-x,1-y,2-z  =      2_667 Check
PLAT911_ALERT_3_C Missing FCF Refl Between Thmin & STh/L=    0.600        121 Report
```

Alert level G

```
FORMU01_ALERT_2_G  There is a discrepancy between the atom counts in the
            _chemical_formula_sum and the formula from the _atom_site* data.
            Atom count from _chemical_formula_sum:C23.5 H22 Co1 N2 O8 S1
            Atom count from the _atom_site data:  C23.5 H21.5 Co1 N2 O8 S1
CELLZ01_ALERT_1_G Difference between formula and atom_site contents detected.
CELLZ01_ALERT_1_G WARNING: H atoms missing from atom site list. Is this intentional?
           From the CIF: _cell_formula_units_Z    2
           From the CIF: _chemical_formula_sum  C23.50 H22 Co N2 O8 S
           TEST: Compare cell contents of formula and atom_site data

           atom    Z*formula  cif sites diff
           C         47.00     47.00    0.00
           H         44.00     43.00    1.00
           Co         2.00      2.00    0.00
           N          4.00      4.00    0.00
           O         16.00     16.00    0.00
           S          2.00      2.00    0.00
PLAT002_ALERT_2_G Number of Distance or Angle Restraints on AtSite          2 Note
PLAT004_ALERT_5_G Polymeric Structure Found with Maximum Dimension          2 Info
PLAT007_ALERT_5_G Number of Unrefined Donor-H Atoms ..............          3 Report
PLAT045_ALERT_1_G Calculated and Reported Z Differ by a Factor ...       0.50 Check
PLAT068_ALERT_1_G Reported F000 Differs from Calcd (or Missing)...     Please Check
PLAT154_ALERT_1_G The s.u.'s on the Cell Angles are Equal ..(Note)      0.005 Degree
PLAT172_ALERT_4_G The CIF-Embedded .res File Contains DFIX Records          1 Report
PLAT300_ALERT_4_G Atom Site Occupancy of C24        Constrained at        0.5 Check
PLAT300_ALERT_4_G Atom Site Occupancy of H24A       Constrained at        0.5 Check
```

```
PLAT300_ALERT_4_G Atom Site Occupancy of H24B       Constrained at        0.5 Check
PLAT300_ALERT_4_G Atom Site Occupancy of H24C       Constrained at        0.5 Check
PLAT300_ALERT_4_G Atom Site Occupancy of O9         Constrained at        0.5 Check
PLAT300_ALERT_4_G Atom Site Occupancy of H9C        Constrained at        0.5 Check
PLAT300_ALERT_4_G Atom Site Occupancy of H9D        Constrained at        0.5 Check
PLAT302_ALERT_4_G Anion/Solvent/Minor-Residue Disorder (Resd  2  )        50% Note
PLAT302_ALERT_4_G Anion/Solvent/Minor-Residue Disorder (Resd  3  )       100% Note
PLAT794_ALERT_5_G Tentative Bond Valency for Co        (II)      .       1.93 Info
PLAT804_ALERT_5_G Number of ARU-Code Packing Problem(s) in PLATON           4 Info
PLAT860_ALERT_3_G Number of Least-Squares Restraints .............          1 Note
PLAT883_ALERT_1_G No Info/Value for _atom_sites_solution_primary .     Please Do !
PLAT912_ALERT_4_G Missing # of FCF Reflections Above STh/L=  0.600         24 Note
PLAT978_ALERT_2_G Number C-C Bonds with Positive Residual Density.          1 Info
```

```
   0 ALERT level A = Most likely a serious problem - resolve or explain
   0 ALERT level B = A potentially serious problem, consider carefully
  12 ALERT level C = Check. Ensure it is not caused by an omission or oversight
  25 ALERT level G = General information/check it is not something unexpected

   7 ALERT type 1 CIF construction/syntax error, inconsistent or missing data
  10 ALERT type 2 Indicator that the structure model may be wrong or deficient
   5 ALERT type 3 Indicator that the structure quality may be low
  11 ALERT type 4 Improvement, methodology, query or suggestion
   4 ALERT type 5 Informative message, check
```

It is advisable to attempt to resolve as many as possible of the alerts in all categories. Often the minor alerts point to easily fixed oversights, errors and omissions in your CIF or refinement strategy, so attention to these fine details can be worthwhile. In order to resolve some of the more serious problems it may be necessary to carry out additional measurements or structure refinements. However, the purpose of your study may justify the reported deviations and the more serious of these should normally be commented upon in the discussion or experimental section of a paper or in the "special_details" fields of the CIF. checkCIF was carefully designed to identify outliers and unusual parameters, but every test has its limitations and alerts that are not important in a particular case may appear. Conversely, the absence of alerts does not guarantee there are no aspects of the results needing attention. It is up to the individual to critically assess their own results and, if necessary, seek expert advice.

Publication of your CIF in IUCr journals

A basic structural check has been run on your CIF. These basic checks will be run on all CIFs submitted for publication in IUCr journals (*Acta Crystallographica*, *Journal of Applied Crystallography*, *Journal of Synchrotron Radiation*); however, if you intend to submit to *Acta Crystallographica Section C* or *E* or *IUCrData*, you should make sure that full publication checks are run on the final version of your CIF prior to submission.

Publication of your CIF in other journals

Please refer to the *Notes for Authors* of the relevant journal for any special instructions relating to CIF submission.

PLATON version of 03/05/2019; check.def file version of 29/04/2019

Datablock 1 - ellipsoid plot

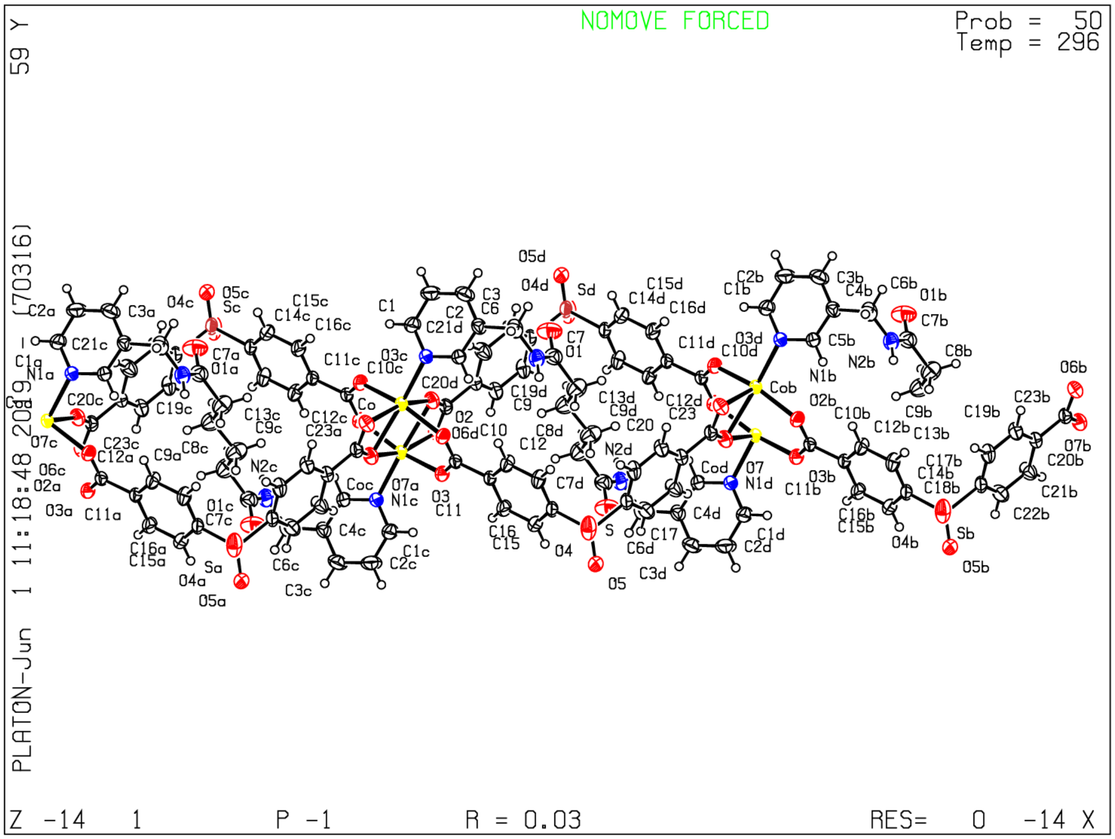

Datablock 2 - ellipsoid plot

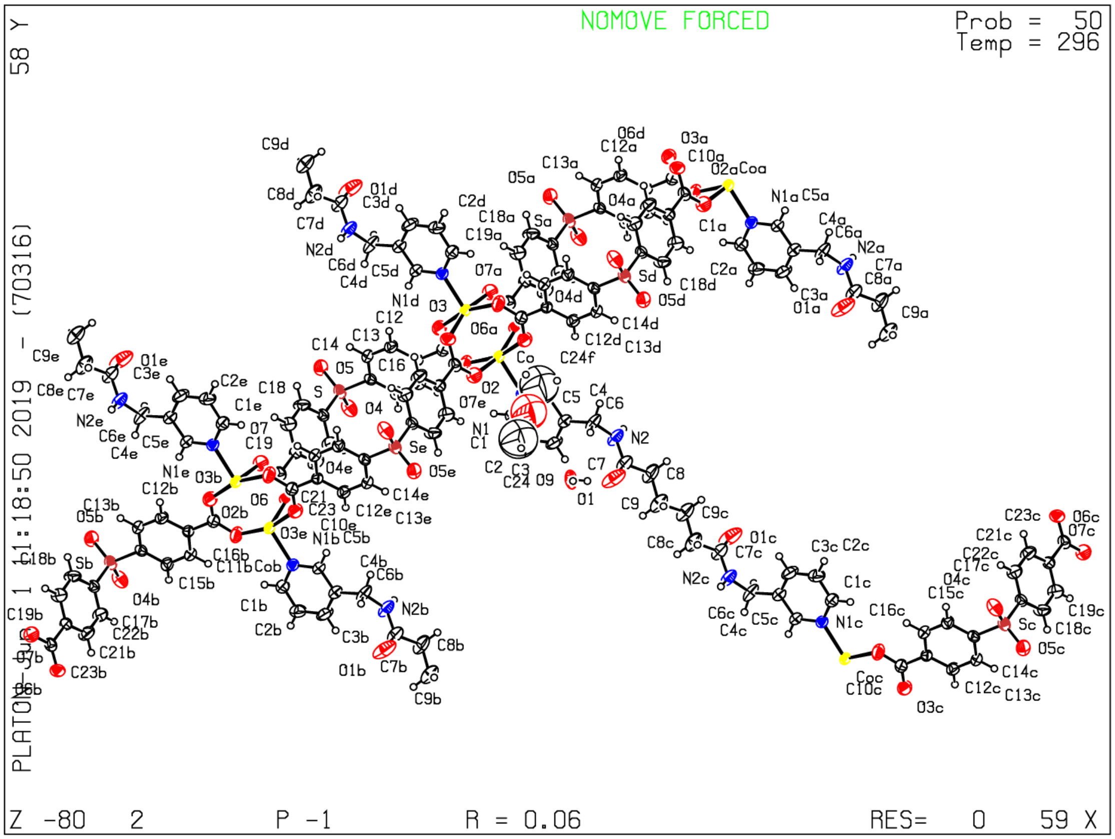

Supplement: Supplementary file 1 [file molecules-25-00201-s001.zip › checkcif.pdf]
