# Supplementary material for: A Pair of CoII Supramolecular Isomers Based on Flexible Bis-Pyridyl-Bis-Amide and Angular Dicarboxylate Ligands
Source: Molecules. 2020 Jan 3;25(1):201. doi: 10.3390/molecules25010201 (PMC6982743; doi:10.3390/molecules25010201)

Supplementary Materials

A Pair of Co(Ii) Supramolecular Isomers Based on Flexible Bis-Pyridyl-Bis-Amide and Angular Dicarboxylate Ligands

Pradhumna Mahat Chhetri,^1^ Ming-Hao Wu,^2^ Chou-Ting Hsieh,^2^ Xiang-Kai Yang,^2^ Chen-Ming Wu,^3^ En-Che Yang, ^3,^* Chih-Chieh Wang^4,^* and Jhy-Der Chen ^2,^*

**
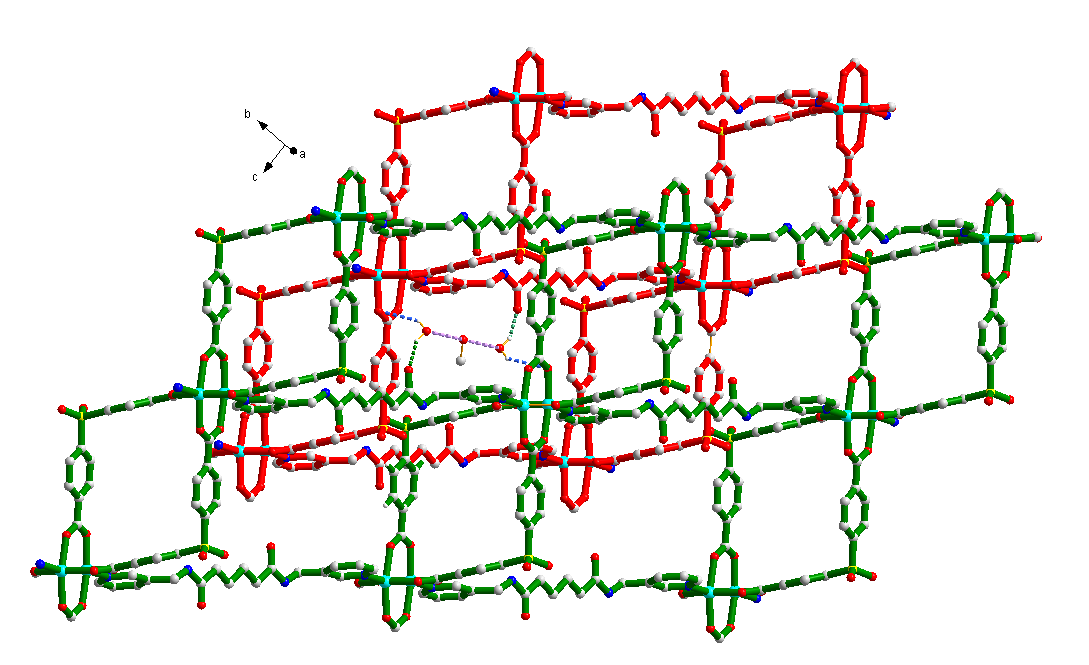
**

**Figure 1.** A drawing showing the hydrogen bondings.

**
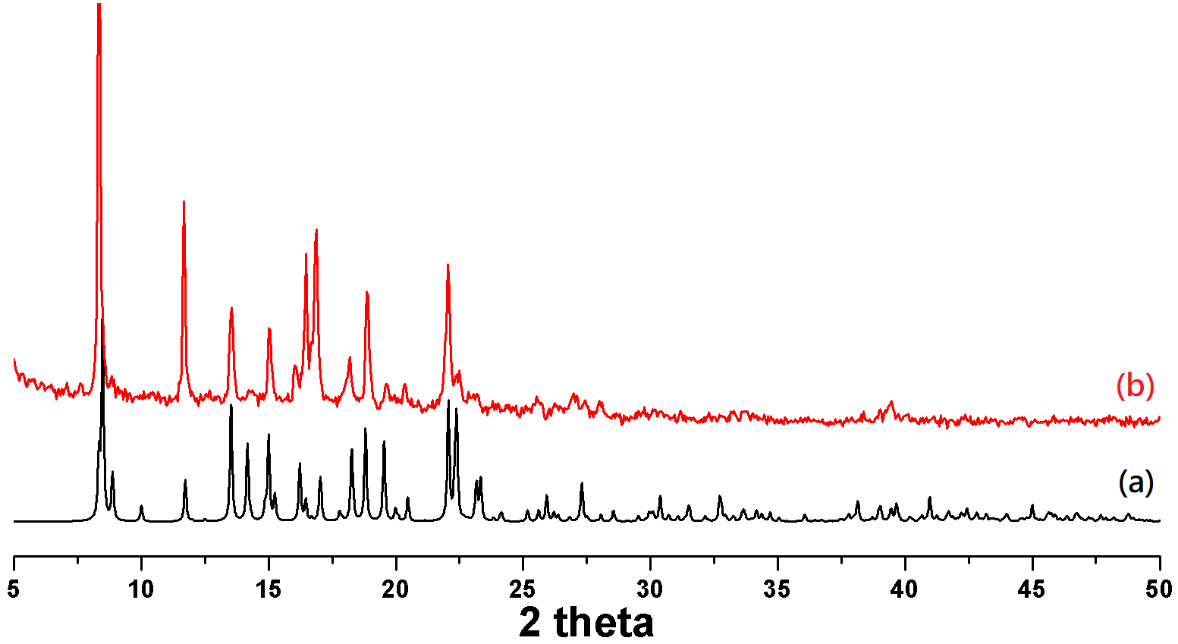
**

**Figure S2.** Powder X-ray patterns of **1**. (a) simulation and (b) experiment.


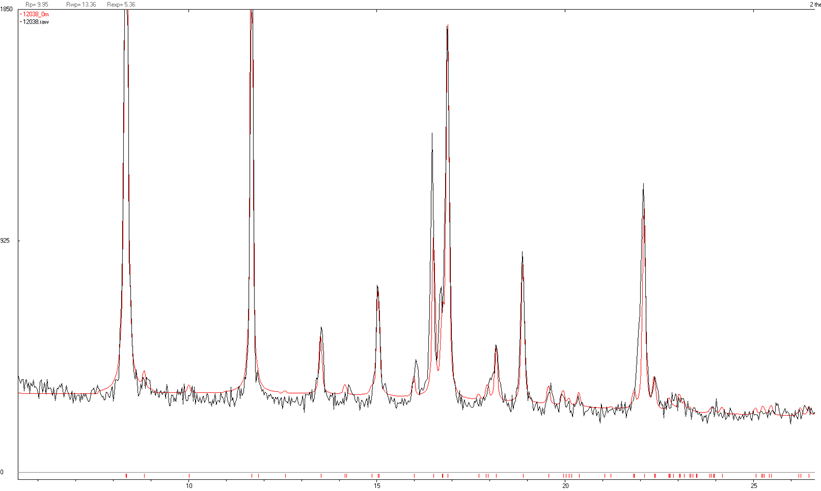


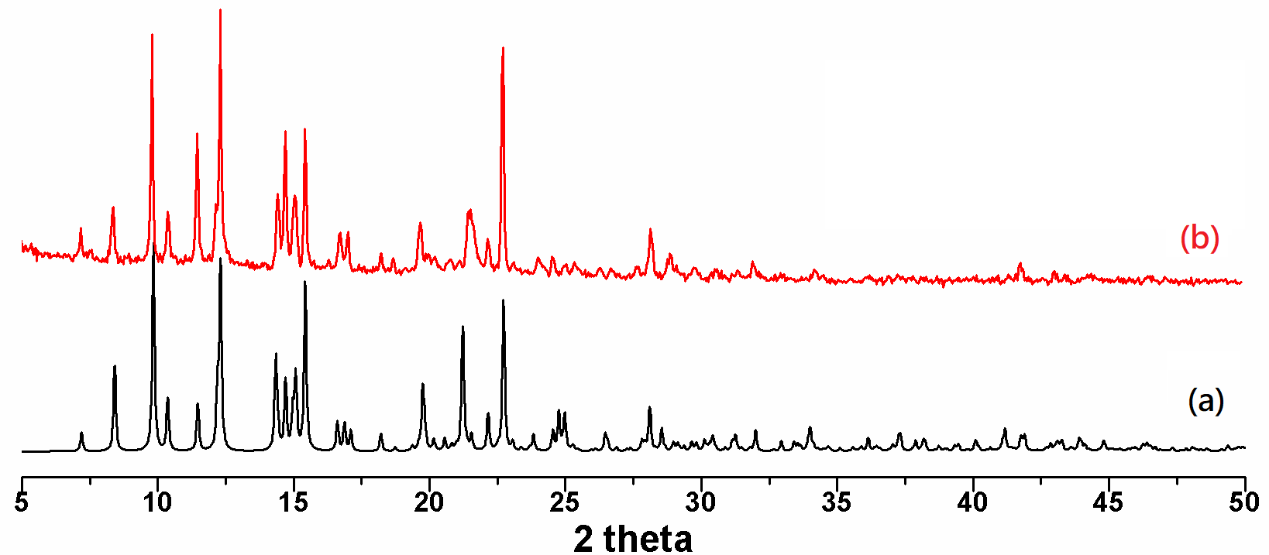


**Figure S3.** Powder X-ray patterns of **2**. (a) simulation and (b) experiment.


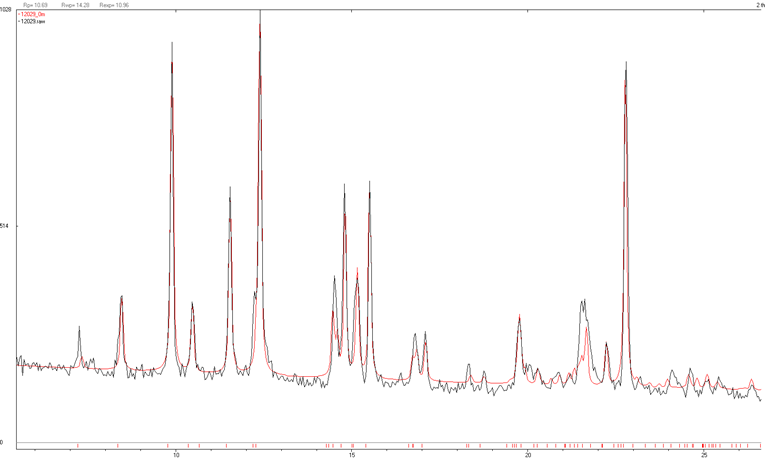

Supplement: Supplementary file 1 [file molecules-25-00201-s001.zip › supplementary_material.docx]
